# Ultrastable, cationic three-dimensional lead bromide frameworks that intrinsically emit broadband white-light†
†Electronic supplementary information (ESI) available: Experimental details and additional characterization. CCDC 1575434–1575436. For ESI and crystallographic data in CIF or other electronic format see DOI: 10.1039/c7sc04118g


**DOI:** 10.1039/c7sc04118g

**Published:** 2017-12-19

**Authors:** Chengdong Peng, Zewen Zhuang, Huimin Yang, Guiyang Zhang, Honghan Fei

**Affiliations:** a Shanghai Key Laboratory of Chemical Assessment and Sustainability , School of Chemical Science and Engineering , Tongji University , 1239 Siping Rd. , Shanghai 200092 , P. R. China . Email: fei@tongji.edu.cn

## Abstract

Herein, we report the unusual broadband white-light emission as an intrinsic property from two cationic lead bromide frameworks. This is the first time that the metal halide materials adopting a purely inorganic positively-charged three-dimensional (3D) topology have been synthesized, thus affording highly distorted Pb^II^ centers. The single-component white-light emitters achieve an external quantum efficiency of up to 5.6% and a correlated color temperature of 5727 K, producing typical white-light close to that of fluorescent light sources. Unlike the air/moisture-sensitive 3D organolead halide perovskites, our cationic materials are chemically “inert” over a wide range of pH as well as aqueous boiling condition. Importantly, these long-sought ultrastable lead halide materials exhibit undiminished photoluminescence upon continuous UV-irradiation for 30 days under atmospheric condition (∼60% relative humidity, 1 bar). Our mechanistic studies indicate the broadband emission have contributions from the self-trapped excited states through electron-vibrational coupling in the highly deformable and anharmonic lattice, as demonstrated by variable-temperature photoluminescence/absorption spectra as well as X-ray crystallography studies. The chemical robustness and structural tunability of the 3D cationic bromoplumbates open new paths for the rational design of hybrid bulk emitters with high photostability.

## Abstract 2

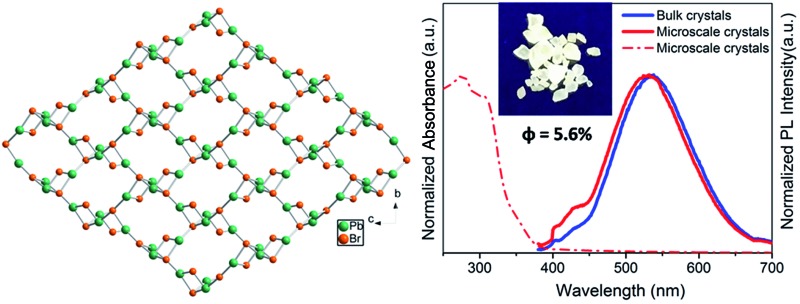
We have discovered the first two lead halide materials adopting a purely cationic inorganic 3D topology. The highly distorted Pb^II^ centers afford strong electron–phonon coupling in a deformable lattice and unusual broadband white-light emission as an intrinsic property.

## Introduction

Solid-state lighting, an energy-saving alternative technology to conventional lighting sources, has attracted increasing attention in recent years.^1^ Among them, realizing white-light luminescence from light-emitting diodes (LEDs) is of particular interest for general illumination applications.^2^–^4^ A typical white-light LED device includes a blend of red, green and blue (RGB) LEDs or coating a blue (or near UV) LED with a yellow phosphor (or a mixture of RGB phosphors).^5^–^7^ These multi-color and/or multi-component strategies suffer from a variety of inevitable drawbacks, such as efficiency losses arising from self-absorption and long-term instability due to different degradation rates of the phosphors.^8^,^9^ Thus, a single-component broadband white-light emitter covering the entire visible spectrum is an attractive and challenging target in white-light LED research.^10^–^12^ However, very few known examples of single-source phosphors intrinsically emit white-light luminescence, thus hindering rational synthetic design.^3^,^5^,^13^

Hybrid inorganic–organic lead halide perovskites containing vertex-sharing metal halide octahedra are an emerging class of photoactive materials, which have promising applications in photovoltaics and light-emitting devices.^14^,^15^ Among them, two-dimensional (2D) organolead halide perovskites usually exhibit narrow-band photoluminescence owing to their flat (100)-oriented layers and large exciton binding energy.^16^ Recently, a few instances of (110)-oriented 2D perovskites showed broadband photoemission as an intrinsic property, presumably ascribed to the formation of self-trapped excited states (*e.g.* Pb_2_^3+^, Pb^3+^, X_2_^–^) (X = Cl, Br, I).^17^–^24^ Lowering the dimensionality of lead halide facilitates the self-trapping process and enhances the photoluminescence quantum efficiency (PLQE), albeit with a sacrifice in structural stability and photoluminescence tunability.^25^–^29^ The air/moisture-sensitive nature of organolead halide perovskites results in a gradual decrease of photoemission intensity upon UV-irradiation in air, thus hindering their industrial applications in LED technology.^30^–^32^ Therefore, the development of chemically ultrastable lead halide materials with efficient, tunable and broadband white-light emission is crucial to extend their photoactive applications.

Inorganic extended frameworks usually adopt a neutral or anionic inorganic host, including zeolites, aluminophosphates and metal halide perovskites.^33^–^35^ Purely inorganic structures bearing an overall positive charge are very rare, presenting merely <0.1% of over 150 000 crystal structures in the inorganic crystal structure database (ICSD). Layered double hydroxides are a widely studied class of cationic 2D inorganic materials, possessing trivalent-ion-substituted brucite layers intercalated with charge-balancing anions. Other examples include francisite minerals (Cu_2_BiSe_2_O_8_X, X = F, Cl, Br, I) and their derivatives,^36^,^37^ as well as layered heavy p-block hydroxides and fluorides.^38^–^42^ Until now, only two synthetic examples of purely inorganic cationic three-dimensional (3D) frameworks have been reported, namely a thorium borate and an ytterbium oxyhydroxide, respectively.^43^,^44^ Among the few examples of 3D lead halide inorganic frameworks, none of them bear a positively charge.^45^,^46^

Very recently, we reported a class of 2D (layered) cationic lead halide materials with high chemical resistance and high photoluminescence quantum efficiency.^47^ Herein, we report the synthesis, crystal structures and broadband photoluminescence of the first two 3D cationic metal halide frameworks, [Pb_2_Br_2_][O_2_C(CH_2_)_4_CO_2_] and [Pb_3_Br_4_][O_2_C(CH_2_)_2_CO_2_] (which we denote as TJU-6 for [Pb_2_Br_2_]^2+^ and TJU-7 for [Pb_3_Br_4_]^2+^, TJU = Tongji University). The unique positively charged 3D lead bromide networks define the arrays of unidimensional channels, in which reside the bridging organic anions. Intriguingly, both materials are intrinsically bulk white-light emitters spanning the entire visible-light spectrum. In contrast to lead halide perovskite and other hybrid bulk emitters, our cationic materials are essentially unaffected upon near-UV-irradiation (365 nm) for 30 days under ambient condition (∼60% relative humidity, 1 bar). The temperature-dependent photophysical studies (*e.g.* UV-vis absorption spectra and photoluminescence spectra) and X-ray crystallography studies attribute the broad emission to the self-trapped states from electron-vibrational coupling in the strongly deformable and anharmonic lattice.

## Results and discussion

**Fig. 1:**
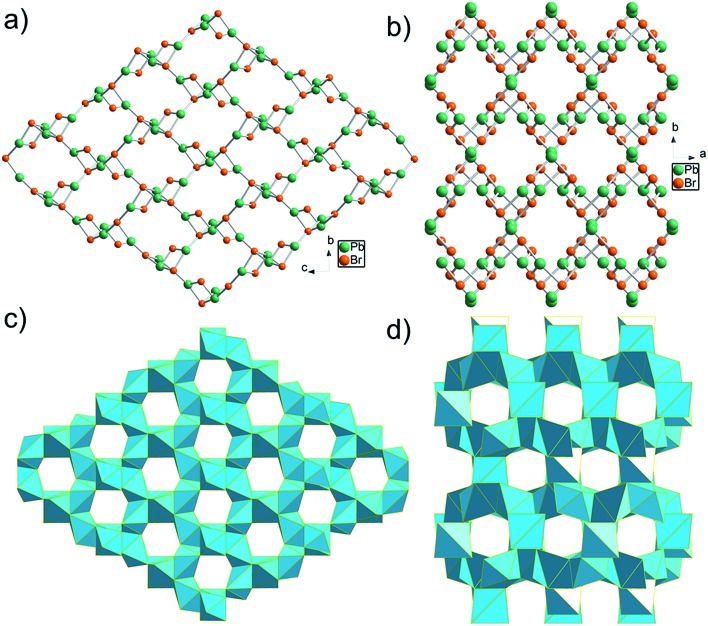
Crystallographic view of TJU-6 and TJU-7: (a, b) ball-and-stick view of TJU-6 (a) and TJU-7 (b), with organic anions omitted for clarity. (c, d) Polyhedral view of TJU-6 (c) and TJU-7 (d). Cyan polyhedra represents PbBr_3_O_3_ in TJU-6 and PbBr_4_O_2_/PbBr_2_O_2_ in TJU-7, respectively. Carbon and hydrogen atoms are omitted for clarity.

Hydrothermal reaction of PbBr_2_, adipic acid disodium salt (NaO_2_C(CH_2_)_4_CO_2_Na), and perchloric acid afforded colorless block-shaped crystals of TJU-6 (Fig. S1a†). Specifically, slow-cooling of the autoclaves at the rate of 10 °C h^–1^ after the static heating is necessary to obtain high phase purity of TJU-6. In addition, perchloric acid was discovered to tune the pH as well as act as a stabilizer, like the role of fluoride ion in zeolite synthesis.^48^ X-Ray crystallography reveals that TJU-6 is crystallized in the highly symmetric tetrahedral *P*4_1_2_1_2 space group (Table S1†). TJU-6 consists of edge- and vertex-sharing PbBr_3_ units extending in three dimensions (Fig. 1a and S2†). Adipates covalently bridge and crosslink the inorganic extended connectivity, further enhancing the structural stability that will be discussed later. The crystallographically independent Pb^II^ atom occupies a highly distorted octahedral geometry in TJU-6, having three bridging bromines and three oxygens from two adipate anions (Fig. S3† inset). In metal halide materials (*e.g*. organolead halide perovskites), there is a strong correlation between the distortion of Pb^II^ centers and the self-trapped excitons from short-range electron–lattice interactions.^14^,^18^,^19^,^23^ Noting the Pb–O bond length is obviously shorter than Pb–Br, we sought to use the octahedral angle variance *σ*_oct_^2^ to quantitatively evaluate the deformation of *O*_h_ symmetry in each PbBr_3_O_3_ octehedra:^49^1
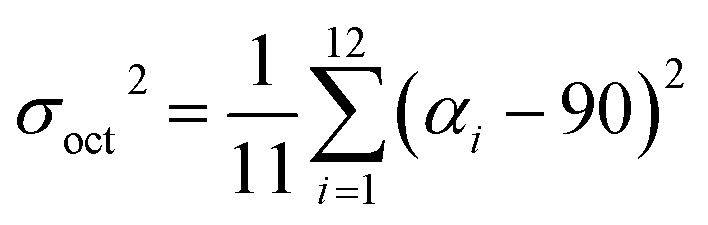
where *α*_*i*_ are the X–Pb–X (X = O/Br) angles. The PbBr_3_O_3_ units in TJU-6 have a large structural distortion (*σ*_oct_^2^ = 735.0), which probably result from the inert pair of s^2^ electrons as well as the different electronegativity of oxygen and bromine atoms.

The edge-sharing PbBr_3_O_3_ octahedra in TJU-6 are connected in both of the crystallographically (100) and (010) planes, defining the honeycomb arrays of 6-membered ring channels along the *a*- and *b*-axis, respectively (Fig. 1c and S3†). Adipates covalently bridge the lead centers within the hexagon-shaped channels, and the Pb–O bond lengths (2.505–2.664 Å) are well within the accepted Pb–O covalent range. The two crystallographically independent Br atoms are either vertex-bridging (μ_2_-Br) or quadruply bridging (μ_4_-Br) in a highly distorted tetrahedral geometry. Overall, the high-coordinated bridging Br atoms and the low-coordinated Pb centers collectively attribute to the cationic feature of a rare 3D metal halide topology.

Synthesis of the cationic 3D bromoplumbate framework was successfully extended to a more compact-packing inorganic topology *via* employing a shorter α,ω-alkanecarboxylate as the anionic structure-directing agent. Colorless block-shaped crystals of TJU-7 (Fig. S1b†) were prepared using succinate in place of adipate under otherwise identical synthetic conditions. TJU-7 crystallizes in the orthorhombic crystal system with *Pbcn* space group (Table S2†). The structure of TJU-7 consists of cationic [PbBr]^+^ chains along the *c*-axis and Pb_2_Br_4_ units vertex-bridge the adjacent chains (Fig. 1b and S4†). The crystallographically independent Pb^II^ center in the cationic chains adopts a distorted octahedral geometry, as for Pb^II^ in TJU-6. According to octahedral angle variance (*σ*_oct_^2^) calculation (eqn (1)), the structural distortion of TJU-7 (*σ*_oct_^2^ = 783) is close to that of TJU-6 (*σ*_oct_^2^ = 735). The other crystallography independent Pb^II^ centers are in the Pb_2_Br_4_ pillars, which occupy a distorted tetrahedral geometry (Fig. S5† inset). The corner-sharing Pb_2_Br_4_O_2_ octahedra and vertex-sharing Pb_2_Br_2_O_2_ tetrahedra define 8-membered ring channels along the *c*-axis, in which reside the crosslinked succinates (Fig. 1d and S5†). It is worth noting that the shorter α,ω-alkanecarboxylate results in the inorganic framework of TJU-7 adopting a dense topology as well as a lower stoichiometric ratio of Pb : Br (3 : 4), compared to 1 : 1 in the open framework of TJU-6

Non-perovskite metal-halide hybrid materials usually have a low-dimensional inorganic extended structure (1D or 2D). In addition, all of the previously reported 3D lead halide examples occupy an anionic inorganic network hosting organoammonium cations.^45^,^46^,^50^,^51^ Overall, TJU-6 and TJU-7 are the first two lead halide materials with a cationic 3D M–X–M (M = metal, X = halogen) connectivity that have been unambiguously identified by single-crystal X-ray crystallography.

**Fig. 2:**
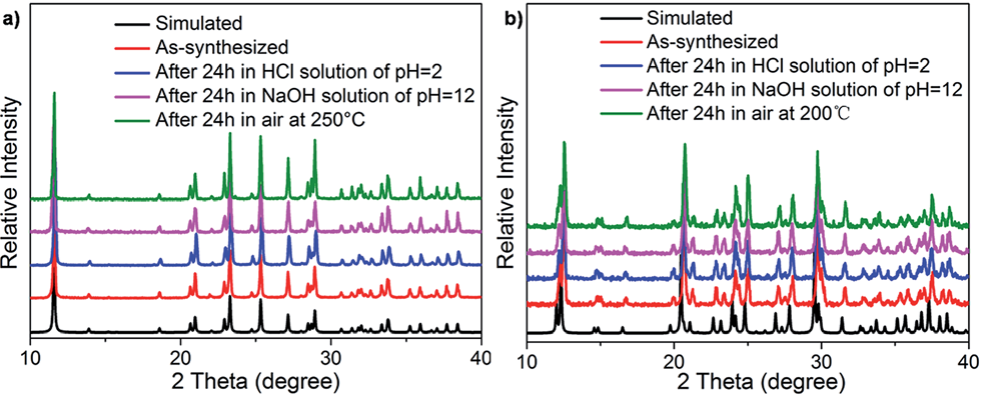
PXRD of TJU-6 (a) and TJU-7 (b) before and after heat or chemical treatment.

The high yield and phase purity of TJU-6 and TJU-7 was evidenced by Fourier-transform infrared spectroscopy (FTIR), elemental analysis and powder X-ray diffraction (PXRD), which matches well with the theoretical patterns simulated from single-crystal data (Fig. 2, S6 and S7†). Unlike organoammonium cations in perovskites, the anionic structure-directing agents (*e.g.* α,ω-alkanecarboxylate) covalently crosslink the metal centers within the pore channels of the cationic inorganic host. This structural feature plays a significant role to afford the chemical “inertness” of the resulting lead bromide materials. Stability tests were performed by treating the materials in water, ethanol, HCl solution (pH = 2), and NaOH solution (pH = 12) for 24 h. PXRD of the post-treated TJU-6 and TJU-7 remained intact, confirming the well-retained cationic topology (Fig. 2). In addition, no apparent loss in mass was observed during the chemical treatment, further proving the chemical inertness of TJU-6 and TJU-7. Moreover, thermogravimetric analysis (TGA) and *ex situ* thermodiffraction indicate that TJU-6 and TJU-7 are thermally stable up to 250 °C under air (Fig. 2, S8 and S9†). Overall, our cationic 3D bromoplumbate materials strikingly push forward the chemical-resistance and related photoactive applications of organolead halide hybrid materials.

**Fig. 3:**
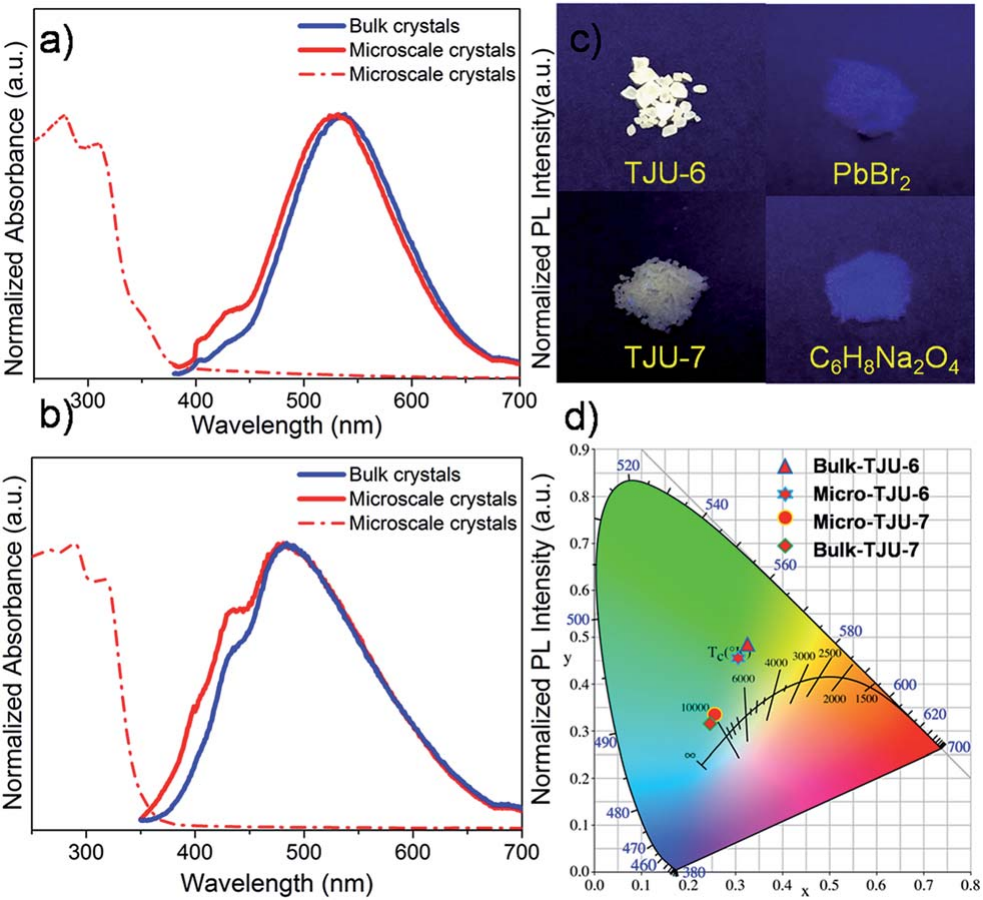
(a, b) Emission spectra of the cm-sized bulk crystals (blue) and μm-sized microscopic crystals (red) of TJU-6 (excited at 360 nm) and TJU-7 (excited at 370 nm). (c) Photoimages of TJU-6, TJU-7, PbBr_2_ and adipate under 4 W 365 nm UV-light. (d) CIE chromaticity coordinates of TJU-6 and TJU-7.

Given the high robustness and lattice deformation of TJU-6 and TJU-7, we sought to investigate their photoluminescent properties (Table 1). Both materials show a typical semiconductive properties with an optical bandgap of 3.70 eV (335 nm) for TJU-6 and 3.50 eV (354 nm) for TJU-7, respectively, evidenced by UV-vis absorption spectra (Fig. 3a and b). The large band-gaps probably result from the open inorganic framework of [PbBr]^+^ and [Pb_3_Br_4_]^3+^, which leads to an increase in the bandgap and the energy level of conduction band.^52^ In addition, excitonic features (the shoulder peak at 3.99 eV for TJU-6 and 3.90 eV for TJU-7) are observed in the optical absorption spectra. The less-defined excitonic peak is largely ascribed to the moderate exciton binding energy (290 meV) from the absorption spectra of TJU-6 at 103 K (Fig. S10†).

**Table 1:** Photophysical properties of bulk TJU-6 and TJU-7 crystals^*a*^

Material	*λ*_abs_/nm	*λ*_ex_/nm	*λ*_em_/nm	FWHM/nm	*φ* (%)	*τ*_av_/ns
TJU-6	335	360	530	124	5.6	1.7
TJU-7	354	370	480	166	1.8	1.6

^*a*^ *λ*_abs_ is the wavelength at absorbance maximum; *λ*_ex_ is the excitation wavelength; *λ*_em_ is the wavelength at the emission maxima; *φ*; is the external photoluminescence quantum efficiency; *τ*_av_ is the PL lifetime.

**Fig. 4:**
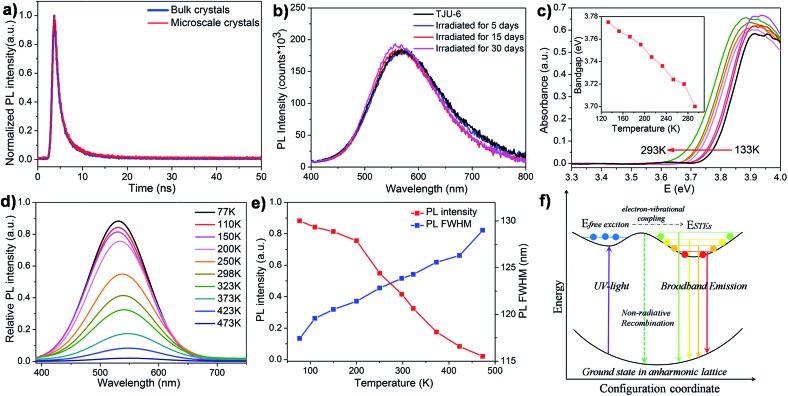
(a) The photoluminescence decay of TJU-6 (measured at 537 nm) at room temperature. (b) Photoemission spectra of TJU-6 before and after continuous UV-irradiation (4 W, 365 nm) under atmospheric condition (∼60% relative intensity, 1 bar, room temperature). (c) Temperature-dependent UV-vis diffuse reflectance micro-spectrum of TJU-6 from 133 to 293 K. (d) Temperature-dependent photoemission spectra of TJU-6 from 77 K to 473 K. (e) Integrated photoluminescence intensity (red) and FWHM (blue) as a function of temperature. (f) Schematic summarizing the main mechanism responsible for broadband emissions in the cationic lead bromide frameworks.

Interestingly, colorless block-shaped single crystals of TJU-6 show a pronounced white-light emission upon irradiation with a 4 W, 365 nm UV-lamp (Fig. 3c). The photoemission spectra (under 360 nm excitation) of TJU-6 demonstrates the unusual broadband photoluminescence spanning the entire visible spectrum (400–700 nm). The strongly Stokes-shifted broadband emission of TJU-6 has a maximum at 530 nm with a large FWHM up to 124 nm (0.56 eV), significantly overcoming the self-absorption problems in the near-UV region. In addition, μm-sized particles of TJU-6 were prepared *via* manual grinding, and showed nearly identical photoemission spectra (Fig. 3 and S11a†). These results confirm that the broadband emission arises from the bulk materials instead of from surface defect sites (which often contribute to the photoluminescence of lead halide perovskite nanocrystals^53^,^54^). The high-energy shoulder at 420 nm is more evident in microscale crystals, likely due to the strong self-adsorption in cm-sized single crystals.^25^ Since the two-band emission profiles are similar to those of lead halide perovskites,^17^,^18^ it is reasonable to attribute the high-energy shoulder to free excitons and the low-energy broadband emission to self-trapped excitons. The detailed photoemission mechanism will be discussed later. Time-resolved photoluminescence decay experiments indicate the lifetime of the emission measured at 537 nm is 1.7 ns for TJU-6 (Fig. 4a). The ns-ranged lifetimes are characteristic of fluorescence emission, while the longer lifetime of TJU-6 over the previously reported 2-D lead-based perovskites (*τ*_av_ = 0.23–1.39 ns)^17^–^20^ is presumably attributed to the populated self-trapped excitons. Moreover, the cm-sized single crystals and μm-sized particles of TJU-6 show similar lifetimes, again suggesting the broadband emission as an intrinsic effect. The strongly Stokes-shifted broadband photoemission covering the entire visible-light spectrum has also been observed in TJU-7. A more evident high-energy photoemission shoulder at 415 nm blue-shifts the maximum emission wavelength to 480 nm with a large FWHM up to 166 nm (0.88 eV).

Importantly, no apparent change to the color properties of the broadband emission is observed in TJU-6 when adjusting the excitation from 320 nm to 360 nm, confirming its nature as a broadband monochromatic emitter (Fig. S12†). The Commission International de l’Eclairage (CIE) chromaticity coordinates of the overall TJU-6 emission is determined to be (0.33, 0.48), corresponding to a color temperature of 5727 K (Fig. 3d). The emission is so-called “cold” white-light, and very close to that of conventional fluorescent light sources (∼5100 K). Meanwhile, the μm-sized crystals of TJU-6 exhibit a CIE coordinate of (0.30, 0.45) with a color temperature of 6464 K (Fig. 3d). The external quantum efficiency of the TJU-6 is measured to be 5.6%, which is significantly higher than most white-light emitters based on 2D organolead halide perovskites as well as a 3D anionic lead chloride framework (Table S3†). In addition, the photoemission band and intensity was monitored upon irradiation with a 4 W, 365 UV lamp under atmospheric condition (∼60% relative intensity, 1 bar, room temperature). Intriguingly, the broadband emission is remarkably stable after UV-irradiation in air for 30 days, achieving a substantial advance in hybrid lead halide white-light emitters (Fig. 4b).

The TJU-7 light emission was determined to have CIE chromaticity coordinates of (0.25, 0.32) for bulk cm-sized crystals and (0.25, 0.33) for μm-sized crystal particles, which are all closer to the coordinates of the sunlight source (0.33, 0.33) (Fig. 3d). The bluish white-light emission affords the correlated color temperature of 11 967 K for cm-sized single crystals and 10 640 K for μm-sized particles, respectively. The external PLQE of the TJU-7 was measured to be 1.8% with an expected lower efficiency from the dense [Pb_3_Br_4_]^2+^ framework.

In order to verify the broadband emission of TJU-6 arises from the self-trapped excited states attributable to the lattice deformation, we performed variable-temperature experiments of photoluminescence and UV-vis absorption spectra (Fig. 4d–f). In contrast to intrinsic white-light emitters of organolead halide perovskites,^18^,^55^,^56^ an obvious and gradual blue-shift of the maximum emission wavelength was noticed when the temperature was decreased from 473 K to 77 K (Fig. 4d). The overall photoluminescent peak position was shifted to higher energy by 27 nm (117 meV), while the integrated intensity of the photoemission was increased by 40 times (Fig. 4e). In order to investigate the origin of the photoluminescence blue-shift, we performed variable-temperature UV-vis absorption spectra of TJU-6 from 133 K to 298 K. In agreement with the photoluminescence experiments, a blue-shift of the absorption edge was clearly observed with decreasing temperature (Fig. 4c). Based on these variable-temperature photophysical studies, it is concluded that TJU-6 has a temperature-dependent (133–293 K) optical bandgap. The temperature dependence of the semiconductor bandgap, determined from the UV-vis absorption edge, is usually well described using Varshni’s equation:^57^2
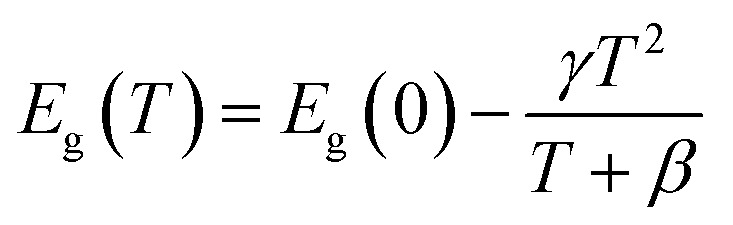
where *E*_g_(*T*) is the energy gap at *T* K; *β* is physically associated with the Debye temperature, and *γ* is the temperature coefficient of the band gap. The best fit is obtained with *E*_g_(0) = 3.815 (5) eV, *γ* = 0.55(5) meV K^–1^, and *β* = 135(35) K (Fig. S13†). The value of *β* = 135(35) K for TJU-6 is within the reasonable range of Debye temperature for PbBr_2_ between 100 and 150 K.^58^–^60^ The temperature coefficient of the band gap (*γ* = 0.55(5) meV K^–1^) is higher than some other typical semiconductors (*e.g.* InN),^61^ suggesting the possible influence of thermal expansion and/or electron–phonon interaction on the band gap in our cationic bromoplumbate materials. A small thermal expansion of the unit cell volume from 1160.5(3) Å^3^ (150 K) to 1166.5(3) Å^3^ (298 K) was observed using single-crystal X-ray crystallography (Tables S1 and S4†). In addition, the anisotropic thermal expansion was noticed with stronger elongation (0.25%) along the *c*-axis than that of the other two directions (0.13% for the *a*- and *b*-axis). Despite the contribution of the anharmonic lattice, the high temperature coefficient of the band gap (*γ* = 0.55(5) meV K^–1^) is largely attributed to the strong electron–phonon coupling.

In addition, broadening of the emission bandwidth was observed from 118 nm to 129 nm as the temperature was increased from 77 K to 493 K, further confirming the electron–phonon interactions (Fig. 4d and e). The temperature dependence of the FWHM in TJU-6 can be described using the following model:^62^

3 *Γ*(*T*) = *Γ*_0_ + *Γ*_LO_(e^*E*_LO_/*k*_B_*T*^ – 1)^–1^ + *Γ*_inh_(e^–*E*_b_/*k*_B_*T*^)

Here, *Γ*_0_ represents the emission FWHM at *T* = 0 K, *E*_LO_ is the energy of the longitudinal optical phonon energy, and *E*_b_ represents the average binding energy of the defect states. *Γ*_LO_ and *Γ*_inh_ give the relative contributions of exciton-phonon coupling and inhomogeneous broadening (induced by trap states), respectively. A fit to the data gives *Γ*_0_ = 219 ± 2 meV, *Γ*_LO_ = 131 ± 7 meV, *Γ*_inh_ = 287 ± 3 meV, *E*_LO_ = 13 ± 5 meV, and *E*_b_ = 12 ± 1 meV (Fig. 4e and S15†). The LO phonon energy obtained from the fitting corresponds to a frequency of ∼110 cm^–1^, which lies well within the range of Pb–Br stretching vibration frequencies from Raman spectroscopy (Fig. S16†). Based on our photophysical mechanistic studies, we confirm that the broadband emission and the self-trapped states of TJU-6 are mainly attributed to the electron–phonon coupling in a strongly deformable 3D lead halide lattice (Fig. 4f).^63^ Recent first-principle calculations of lead halide materials indicate the electron–phonon coupling in a deformable lead halide lattice forms self-trapped excitons (*e.g.* Pb_2_^3+^, Pb^3+^, and Br_2_^–^ species), which act as radiative color centers.^64^,^65^

## Conclusions

We have discovered two hybrid bromoplumbate bulk emitters with an unusual broadband white-light emission spanning the entire visible-light region. These are the first two 3D lead halide frameworks possessing an overall positive charge, thus exhibiting high distortion of Pb^II^ centers and generated self-trapped states. Upon 360 nm excitation, the [PbBr]^+^ exhibit a moderately high PLQE of 5.6%, exceeding most of the perovskite-type white-light emitters (<1%). Importantly, the ultrastable nature of our materials affords undiminished photoemission throughout UV-irradiation for 30 days under air, largely overcoming the chemically labile problems of 3D lead perovskites. Our mechanistic studies confirm that the broadband emission arises from electron–phonon coupling in a strongly deformable and anharmonic lattice, which contributes to the formation of self-trapped states. The long-sought “inert” lead halide materials are amenable to synthetic design for enhancing PLQEs, thus serving as potential alternatives to commercial white-light LED phosphors.

## Conflicts of interest

There are no conflicts to declare.

## Supplementary Material

Supplementary information

Crystal structure data

## References

[cit1] O. I. D. Association, The Promise of Solid State Lighting for General Illumination, US Department of Energy, Washington, DC, 2001.

[cit2] Bergh A., Craford G., Duggal A., Haitz R. (2001). Phys. Today.

[cit3] Wang M. S., Guo S. P., Li Y., Cai L. Z., Zou J.-P., Xu G., Zhou W. W., Zheng F. K., Guo G. C. (2009). J. Am. Chem. Soc..

[cit4] Sava D. F., Rohwer L. E., Rodriguez M. A., Nenoff T. M. (2012). J. Am. Chem. Soc..

[cit5] Zhu X. H., Peng J., Cao Y., Roncali J. (2011). Chem. Soc. Rev..

[cit6] Li Y., Rizzo A., Cingolani R., Gigli G. (2006). Adv. Mater..

[cit7] Anikeeva P. O., Halpert J. E., Bawendi M. G., Bulović V. (2007). Nano Lett..

[cit8] Phillips J. M., Coltrin M. E., Crawford M. H., Fischer A. J., Krames M. R., Mueller-Mach R., Mueller G. O., Ohno Y., Rohwer L. E., Simmons J. A. (2007). Laser Photonics Rev..

[cit9] Ye S., Xiao F., Pan Y., Ma Y., Zhang Q. (2010). Mater. Sci. Eng., R.

[cit10] Shang M., Li C., Lin J. (2014). Chem. Soc. Rev..

[cit11] Kamtekar K. T., Monkman A. P., Bryce M. R. (2010). Adv. Mater..

[cit12] Gather M. C., Köhnen A., Meerholz K. (2011). Adv. Mater..

[cit13] Liu Y., Nishiura M., Wang Y., Hou Z. (2006). J. Am. Chem. Soc..

[cit14] Stranks S. D., Snaith H. J. (2015). Nat. Nanotechnol..

[cit15] Berry J., Buonassisi T., Egger D. A., Hodes G., Kronik L., Loo Y. L., Lubomirsky I., Marder S. R., Mastai Y., Miller J. S. (2015). Adv. Mater..

[cit16] Deschler F., Price M., Pathak S., Klintberg L. E., Jarausch D.-D., Higler R., Hüttner S., Leijtens T., Stranks S. D., Snaith H. J. (2014). J. Phys. Chem. Lett..

[cit17] Dohner E. R., Hoke E. T., Karunadasa H. I. (2014). J. Am. Chem. Soc..

[cit18] Dohner E. R., Jaffe A., Bradshaw L. R., Karunadasa H. I. (2014). J. Am. Chem. Soc..

[cit19] Cortecchia D., Neutzner S., Srimath Kandada A. R., Mosconi E., Meggiolaro D., De Angelis F., Soci C., Petrozza A. (2017). J. Am. Chem. Soc..

[cit20] Mao L., Wu Y., Stoumpos C. C., Wasielewski M. R., Kanatzidis M. G. (2017). J. Am. Chem. Soc..

[cit21] Thirumal K., Chong W. K., Xie W., Ganguly R., Muduli S. K., Sherburne M., Asta M., Mhaisalkar S., Sum T. C., Soo H. S. (2017). Chem. Mater..

[cit22] Smith M. D., Jaffe A., Dohner E. R., Lindenberg A. M., Karunadasa H. I. (2017). Chem. Sci..

[cit23] Li Y., Lin C., Zheng G., Cheng Z., You H., Wang W., Lin J. (2006). Chem. Mater..

[cit24] Li Y., Zheng G., Lin J. (2008). Eur. J. Inorg. Chem..

[cit25] Yuan Z., Zhou C., Tian Y., Shu Y., Messier J., Wang J. C., van de Burgt L. J., Kountouriotis K., Xin Y., Holt E., Schanze K., Clark R., Siegrist T., Ma B. (2017). Nat. Commun..

[cit26] Ma B., Zhou C., Tian Y., Wang M., Rose A., Besara T., Doyle N. K., Yuan Z., Wang J. C., Clark R., Hu Y., Siegrist T., Lin S. (2017). Angew. Chem., Int. Ed..

[cit27] Zhou C., Yuan Z., Tian Y., Lin H., Clark R., Chen B., van de Burgt L. J., Wang J. C., Hanson K. and Meisner Q. J., arXiv:1702.07200, 2017.

[cit28] Saidaminov M. I., Almutlaq J., Sarmah S., Dursun I., Zhumekenov A. A., Begum R., Pan J., Cho N., Mohammed O. F., Bakr O. M. (2016). ACS Energy Lett..

[cit29] Saidaminov M. I., Mohammed O. F., Bakr O. M. (2017). ACS Energy Lett..

[cit30] Mosconi E., Azpiroz J. M., De Angelis F. (2015). Chem. Mater..

[cit31] Misra R. K., Aharon S., Li B., Mogilyansky D., Visoly-Fisher I., Etgar L., Katz E. A. (2015). J. Phys. Chem. Lett..

[cit32] Conings B., Drijkoningen J., Gauquelin N., Babayigit A., D’Haen J., D’Olieslaeger L., Ethirajan A., Verbeeck J., Manca J., Mosconi E. (2015). Adv. Energy Mater..

[cit33] Baerlocher C., McCusker L. B. and Olson D. H., Atlas of zeolite framework types, Elsevier, 2007.

[cit34] Yu J., Xu R. (2006). Chem. Soc. Rev..

[cit35] Saparov B., Mitzi D. B. (2016). Chem. Rev..

[cit36] Millet P., Bastide B., Pashchenko V., Gnatchenko S., Gapon V., Ksari Y., Stepanov A. (2001). J. Mater. Chem..

[cit37] Ok K. M., Halasyamani P. S. (2002). Inorg. Chem..

[cit38] Swanson C. H., Shaikh H. A., Rogow D. L., Oliver A. G., Campana C. F., Oliver S. R. J. (2008). J. Am. Chem. Soc..

[cit39] Fei H. H., Oliver S. R. J. (2011). Angew. Chem., Int. Ed..

[cit40] Fei H. H., Pham C. H., Oliver S. R. J. (2012). J. Am. Chem. Soc..

[cit41] Zhang G., Wei G., Liu Z., Oliver S. R. J., Fei H. (2016). Chem. Mater..

[cit42] Yang H., Fei H. (2017). Chem. Commun..

[cit43] Wang S. A., Alekseev E. V., Juan D. W., Casey W. H., Phillips B. L., Depmeier W., Albrecht-Schmitt T. E. (2010). Angew. Chem., Int. Ed..

[cit44] Goulding H. V., Hulse S. E., Clegg W., Harrington R. W., Playford H. Y., Walton R. I., Fogg A. M. (2010). J. Am. Chem. Soc..

[cit45] Zhang Z. J., Xiang S. C., Guo G. C., Xu G., Wang M. S., Zou J. P., Guo S. P., Huang J. S. (2008). Angew. Chem., Int. Ed..

[cit46] Wang G. E., Xu G., Wang M. S., Cai L. Z., Li W. H., Guo G. C. (2015). Chem. Sci..

[cit47] Zhuang Z., Peng C., Zhang G., Yang H., Yin J., Fei H. (2017). Angew. Chem., Int. Ed..

[cit48] Rogow D. L., Russell M. P., Wayman L. M., Swanson C. H., Oliver A. G., Oliver S. R. J. (2010). Cryst. Growth Des..

[cit49] Robinson K., Gibbs G. V., Ribbe P. H. (1971). Science.

[cit50] Corradi A. B., Ferrari A. M., Pellacani G. C., Saccani A., Sandrolini F., Sgarabotto P. (1999). Inorg. Chem..

[cit51] Martin J. D., Greenwood K. B. (1997). Angew. Chem., Int. Ed. Engl..

[cit52] Zheng N., Bu X., Vu H., Feng P. (2005). Angew. Chem., Int. Ed..

[cit53] Teunis M. B., Lawrence K. N., Dutta P., Siegel A. P., Sardar R. (2016). Nanoscale.

[cit54] Wu H., Xu S., Shao H., Li L., Cui Y., Wang C. (2017). Nanoscale.

[cit55] Hu T., Smith M. D., Dohner E. R., Sher M.-J., Wu X., Trinh M. T., Fisher A., Corbett J., Zhu X. Y., Karunadasa H. I., Lindenberg A. M. (2016). J. Phys. Chem. Lett..

[cit56] Yangui A., Garrot D., Lauret J. S., Lusson A., Bouchez G., Deleporte E., Pillet S., Bendeif E. E., Castro M., Triki S., Abid Y., Boukheddaden K. (2015). J. Phys. Chem. C.

[cit57] Varshni Y. P. (1967). Physica.

[cit58] Sastry P., Mtjlimani B. (1969). Philos. Mag..

[cit59] Barr L., Dawson D. (1971). Proc. Br. Ceram. Soc..

[cit60] Kerssen J., De Gruijter W., Volger J. (1973). Physica.

[cit61] Wu J., Walukiewicz W., Shan W., Yu K., Ager Iii J., Li S., Haller E., Lu H., Schaff W. J. (2003). J. Appl. Phys..

[cit62] Fonoberov V. A., Alim K. A., Balandin A. A., Xiu F., Liu J. (2006). Phys. Rev. B: Condens. Matter Mater. Phys..

[cit63] Song K. S. and Williams R. T., Self-Trapped Excitons, Springer, Berlin, 2nd edn, 1996.

[cit64] Cortecchia D., Yin J., Bruno A., Lo S. Z. A., Gurzadyan G. G., Mhaisalkar S., Bredas J. L., Soci C. (2017). J. Mater. Chem. C.

[cit65] Yin J., Li H., Cortecchia D., Soci C., Bredas J. L. (2017). ACS Energy Lett..

