# Supplementary material for: Ultrastable, cationic three-dimensional lead bromide frameworks that intrinsically emit broadband white-light
Source: Chem Sci. 2017 Dec 19;9(6):1627–33. doi: 10.1039/c7sc04118g (PMC5887861; doi:10.1039/c7sc04118g)
Supplement: Supplementary file 1 [file SC-009-C7SC04118G-s001.pdf]

Electronic Supplementary Material (ESI) for Chemical Science.
This journal is © The Royal Society of Chemistry 2017

Supporting Information for:

Ultrastable, Cationic Three-Dimensional Lead Bromide Frameworks that Intrinsically Emits Broadband White-Light†

*Chengdong Peng,‡ Zewen Zhuang,‡ Huimin Yang, Guiyang Zhang and Honghan Fei**

School of Chemical Science and Engineering, Shanghai Key Laboratory of Chemical Assessment and Sustainability, Tongji University, Shanghai 200092, P. R. China

Corresponding author: fei@tongji.edu.cn

‡These authors contributed equally.

Experimental Section

Reagents.

Lead bromide ($PbBr_2$, 99.0%, Aladdin), adipic acid disodium salt ($NaO_2C(CH_2)_4CO_2Na$, >98.0%, TCI), succinic acid disodium salt ($NaO_2C(CH_2)_2CO_2Na$, >98.0%, TCI) and perchloric acid (70 % in H_2O, SCR) were used as-received for the hydrothermal synthesis of TJU-6 and TJU-7.

Synthesis.

$[Pb_2Br_2][O_2C(CH_2)_4CO_2]$ (TJU-6). A mixture of 0.3670 g $PbBr_2$ (1.0 mmol), 0.3800 g disodium adipic acid (2.0 mmol), 230 μL perchloric acid ($HClO_4$, 2.78 mmol), and 8 mL deionized water were added into a 12 mL Teflon-lined autoclave reactor followed by 15 min stirring for sufficient dispersion. The autoclave was then sealed into a stainless steel vessel and heated at 175 ℃ for 48 h. After incubation, slow-cooling of the autoclaves at the rate of 10 ℃/h is necessary to obtain high phase purity of colorless block-shaped crystals of TJU-6. Colorless crystals of TJU-6 were isolated after vacuum filtration, and rinsed with ethanol and deionized water. Yield: 0.294g (82 % based on total Pb content). The μm-sized microscopic powders of TJU-6 were prepared via manual grinding the bulk crystals. Element analysis: calculated C, 10.03%; H, 1.11%; found C, 11.25%; H, 1.34%.

$[Pb_3Br_4][O_2C(CH_2)_2CO_2]$ (TJU-7). Colorless crystal TJU-7 can be synthesized in the same manner as for TJU-6 but with succinic acid disodium salt in place of adipic acid disodium salt at the same molar ratio. Yield 0.264g (75 % based on total Pb content). The μm-sized microscopic powders of TJU-7 were prepared via manual grinding the bulk crystals. Element analysis: calculated C, 4.54%; H, 0.38%; found C, 4.90%; H, 0.53%.

Single Crystal X-ray Diffraction (SC-XRD). Single crystals of TJU-6 and TJU-7 were chosen for X-ray diffraction experiment under an optical microscope, and

mounted onto a glass fiber. The diffraction data were measured using a Bruker SMART APEX II CCD area detector X-ray diffractometer at 295(2) K or 150(2) K, using a graphite-monochromated Mo-Kα radiation (λ= 0.71073 Å) and operated at 50 kV and 30 mA. The structures were solved by direct methods and expanded routinely. The models were refined by full-matrix least-squares analysis of F^2 against all reflections. All non-hydrogen atoms were refined with anisotropic thermal displacement parameters. Programs used were APEX-II v2.1.4,[S1] SHELXTL v6.14,[S2] and Diamond v3.2e.[S3]

Powder X-ray Diffraction (PXRD).PXRD analysis was performed using a Bruker D8 Advance diffractometer (Cu Kα, λ = 1.5418 Å) operating at 40 kV/40 mA. The diffraction patterns were scanned at ambient temperature, with a scan speed of 1 sec/step, a step size of 0.02 ° in 2θ, and a 2θ range of 5~40 °. Simulated powder patterns were calculated by Mercury software using the crystallographic information file from the single-crystal X-ray experiment.

Element Analysis (EA). EA for C, H were operated on a Varian EL Ш element analyzer.

Fourier-transform infrared (FT-IR) spectrum. FT-IR spectra were recorded using a BRUKER ALPHA spectrophotometer in 4000~400 cm^{-1} region.

Thermogravimetric analysis (TGA). TGA was carried out using a TGA Q500 differential thermal analyzer. The samples were heated in N_2 stream (60 mL/min) from room temperature to 800 ℃ with a heating rate of 10 ℃/min.

Chemical and thermal stability studies. Appropriate amounts of TJU-6 or TJU-7 were added into boiling water, ethanol, a HCl solution (pH=2), and a NaOH solution (pH=12), respectively. After incubation in these solutions for 24 h, PXRD analysis was performed. To test the thermal stability of materials, samples were heated in air

at a temperature of 250 ℃ before performing PXRD measurements.

Optical image. Optical microscope images were obtained using a Nikon ECLPSE LV100NPOL. Photoimages were collected using an OPPO R9S Smartphone.

Optical Absorption Spectroscopy. Optical diffuse reflectance measurements were performed using a Shimadzu UV-2600 UV-VIS spectrometer equipped with an integrating sphere, operating in the 200-1000nm region at room temperature. $BaSO_4$ was used as reference of 100% reflectance for all measurements. The reflectance spectra were converted to absorption spectra according to the equation: A = 2-lg(%T), where A and T are the absorbance and reflectance, respectively.

Steady state photoluminescence. Steady-state photoluminescence spectra of both bulk and microscopic crystals were obtained at room temperature on an Edinburgh Instruments FLS980 spectrophotometer.

Photoluminescence quantum efficiencies (PLQEs). Absolute PLQE measurements of both bulk and microscopic crystals were performed on FLS920 spectrophotometer with an integrating sphere ($BaSO_4$ coating) using single photon counting mode. The focal length of the monochromator was 300mm. Samples were excited at 360 nm (TJU-6) or 370nm (TJU-7) using a 450W Xenon lamp with 3mm excitation slits width and detected by a Hamamatsu R928p photomultiplier tube. The emission was obtained using 0.2nm scan step, 0.2s scan dwell time, and 0.1mmemission slit width. The PLQEs were calculated by the equation:$\varphi = \mathrm{k}_f/\mathrm{k}_a$, in which k_f means the number of emitted photons and k_a means the number of absorbed photons.

Time-resolved photoluminescence. Time-resolved emission data was collected using the FLS980spectrophotometer at room temperature. The average lifetime was obtained from bi-exponential decays according to the equation:

$$\tau_{avg} = \frac{\sum a_i \tau_i^2}{\sum a_i \tau_i} \; i = 1,2$$

Where a_i represents the amplitude of each component and τ_i represents the decay time.

Temperature-dependent photoluminescence. Temperature-dependent emission data was collected using the FLS980 spectrophotometer at a series of temperature from 77K to 330K.

Photostability studies. A 4-W, 365nm lamp was used as the continuous irradiation source to test the photostability of TJU-6. Then, steady-state photoemission measurements were performed for samples that are irradiated for 3, 15 and 30 days in air.

Temperature-dependent UV-Vis diffuse reflectance spectroscopy. The UV-Vis diffuse reflectance micro-spectrum were recorded on a CRIAC 20/30PV Technologoes microspectrophotometer at a series of temperature from 133 K to 293 K. Samples were placed on quartz slides under Krytox oil, and data was collected after optimization of microspectrophotometer.

Supporting Figures and Tables

Table S1. Crystal data and structure refinement for TJU-6 under 295 K.

Empirical formula	$C_3H_4O_2PbBr$
Formula weight	359.16
Crystal system	Tetragonal
space group	$P\ 4_12_12$
Unit cell dimensions	a = 9.0035(13) Å
	b = 9.0035(13) Å
	c = 14.390(2) Å
	β = 90.00 deg
Volume/ $Å^3$, Z	1166.5(3), 8
ρ_{calc} /g cm^{-3}	4.090
μ / mm^{-1}	35.663
F(000)	1240
θ range /deg	3.20 to 27.47
Limiting indices	-10 =< h =< 10
	-10 =< k =< 10
	-17 =< l =< 17
Reflections collected	1026
Independent reflections	3106 [R(int) = 0.0711]
Data / restraints / parameters	1026 / 0 / 45
Goodness-of-fit on F^2	1.053
Final R indices [I>2sigma(I)]	R1 = 0.0588, wR2 = 0.1522
R indices (all data)	R1 = 0.0601, wR2 = 0.1537

$R1=\sum(||F_o|-|F_c||) / \sum|F_o|$; $wR2=\{\sum[w(F_o^2-F_c^2)^2]/\sum[w(F_o^2)]^2\}^{1/2}$.

Table S2. Crystal data and structure refinement for TJU-7 under 295 K.

Empirical formula	$C_4H_4O_4Pb_3Br_4$
Formula weight	1057.27
Crystal system	orthorhombic
space group	Pbcn
Unit cell dimensions	a = 8.2193(7) Å
	b = 14.7180(12) Å
	c = 10.7344(8) Å
	β = 90.00 deg
Volume/ Å^3, Z	1298.56(18), 4
ρ_{calc} /g cm^{-3}	5.408
μ / mm^{-1}	51.096
F(000)	1784
θ range /deg	3.36 to 27.55
Limiting indices	-9 =< h =< 9
	-17 =< k =< 17
	-12 =< l =< 12
Reflections collected	1141
Independent reflections	1738 [R(int) = 0.1061]
Data / restraints / parameters	1141 / 0 / 70
Goodness-of-fit on F^2	1.077
Final R indices [I>2sigma(I)]	R1 = 0.0851, wR2 = 0.2209
R indices (all data)	R1 = 0.0880, wR2 = 0.2268

$R1=\sum(||F_o|-|F_c||) / \sum|F_o|$; $wR2=\{\sum[w(F_o^2-F_c^2)^2]/\sum[w(F_o^2)]^2\}^{1/2}$.

Table S3. A summary of PLQE of our materials and other high-dimensional lead halide peovskites.

Materials	PLQE (%)	Ref
(N-MEDA) $PbBr_4$	0.5-1.5	S4
(EDBE)[$PbCl_4$]	2	S5
(EDBE)[$PbBr_4$]	9	S5
(EDBE)[PbI_4]	0.5	S5
PEPC	<1	S6
(N-MPDA)[$PbBr_4$]	0.5	S7
(H_2DABCO)[Pb_2Cl_6]	2.5	S8
**TJU-6**	**5.6**	**This work**
**TJU-7**	**1.8**	**This work**

Table S4. Crystal data and structure refinement for TJU-6 under 150 K.

Empirical formula	$C_3H_4O_2PbBr$
Formula weight	359.16
Crystal system	Tetragonal
space group	P 4_12_12
Unit cell dimensions	a = 8.9917(8) Å
	b = 8.9917(8) Å
	c = 14.354(3) Å
	β = 90.00 deg
Volume/ $Å^3$, Z	1160.5(3), 8
ρ_{calc} /g cm^{-3}	4.111
μ / mm^{-1}	35.848
F(000)	1240
θ range /deg	3.20 to 24.99
Limiting indices	-10 =< h =< 10
	-10 =< k =< 10
	-17 =< l =< 17
Reflections collected	17075
Independent reflections	1023 [R(int) = 0.093]
Data / restraints / parameters	1023 / 48 / 67
Goodness-of-fit on F^2	1.242
Final R indices [I>2sigma(I)]	R1 = 0.0679, wR2 = 0.2263
R indices (all data)	R1 = 0.0680, wR2 = 0.2265

$R1=\sum(||F_o|-|F_c||) / \sum|F_o|$; $wR2=\{\sum[w(F_o^2-F_c^2)^2]/\sum[w(F_o^2)]^2\}^{1/2}$.

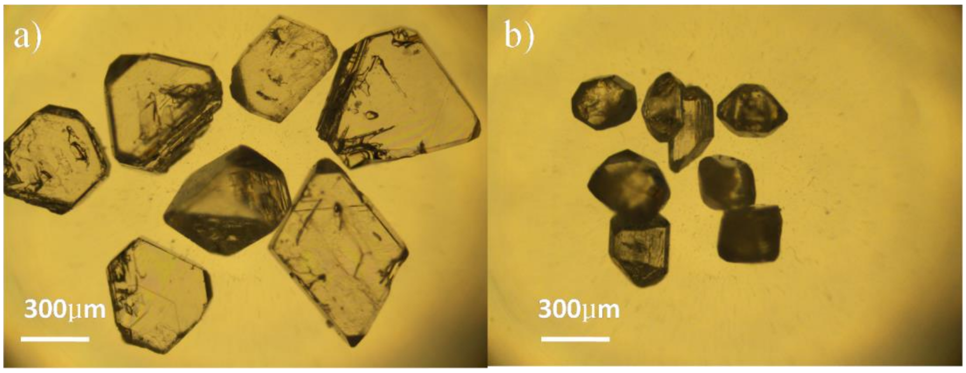

Figure S1. Optical microscope image of TJU-6 (a), and TJU-7 (b)

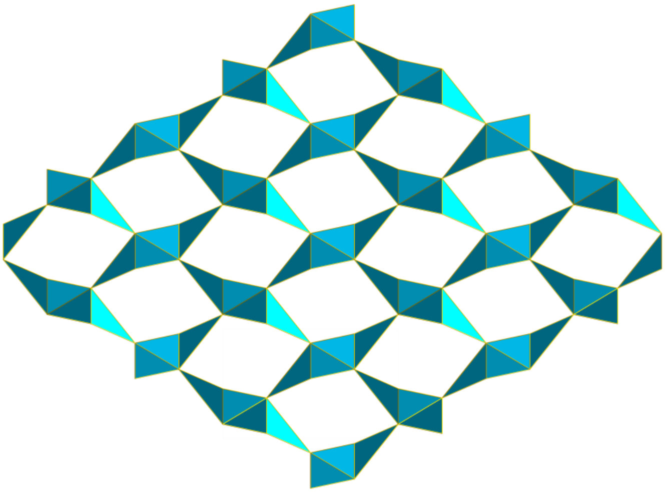

Figure S2. Polyhedral view of the inorganic skeleton of TJU-6

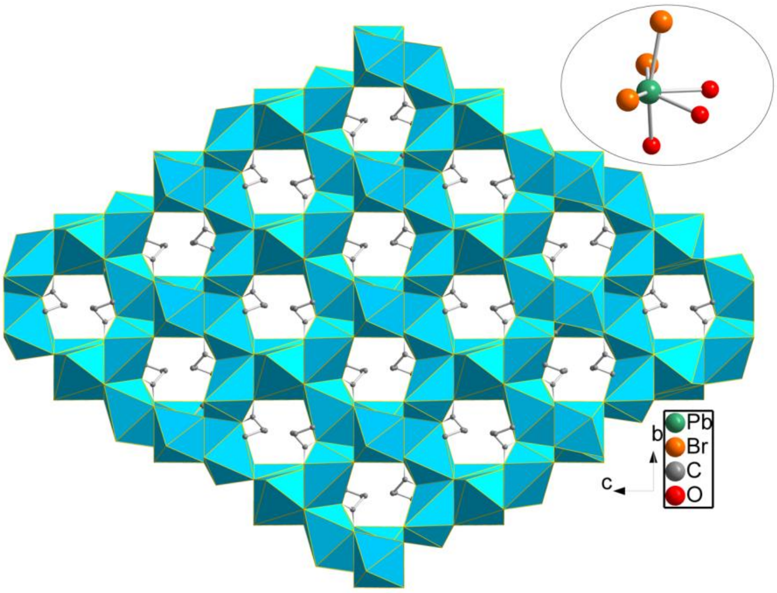

Figure S3. Polyhedral view of the inorganic skeleton of TJU-6 with organic ligands residing in the channels. The inset shows the coordination environment of Pb^{II} center.

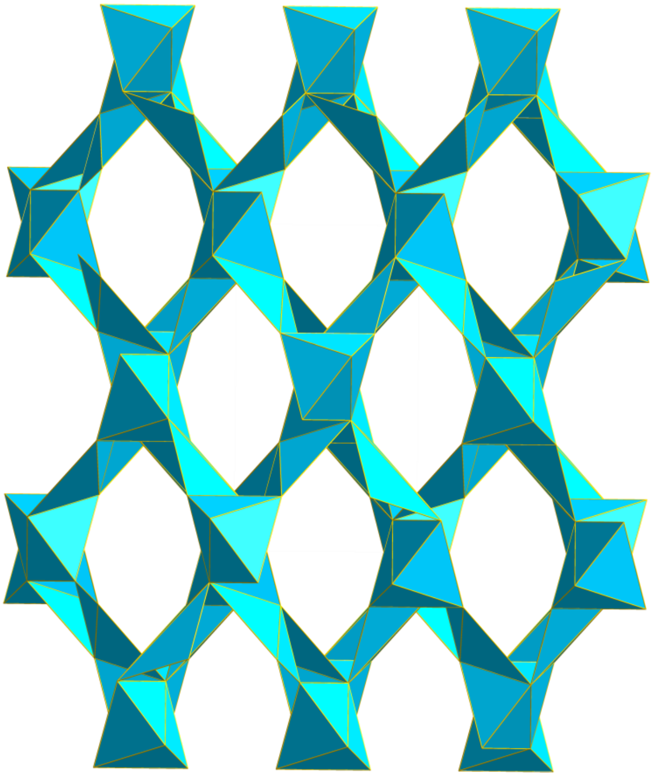

Figure S4. Polyhedral view of the inorganic skeleton of TJU-7.

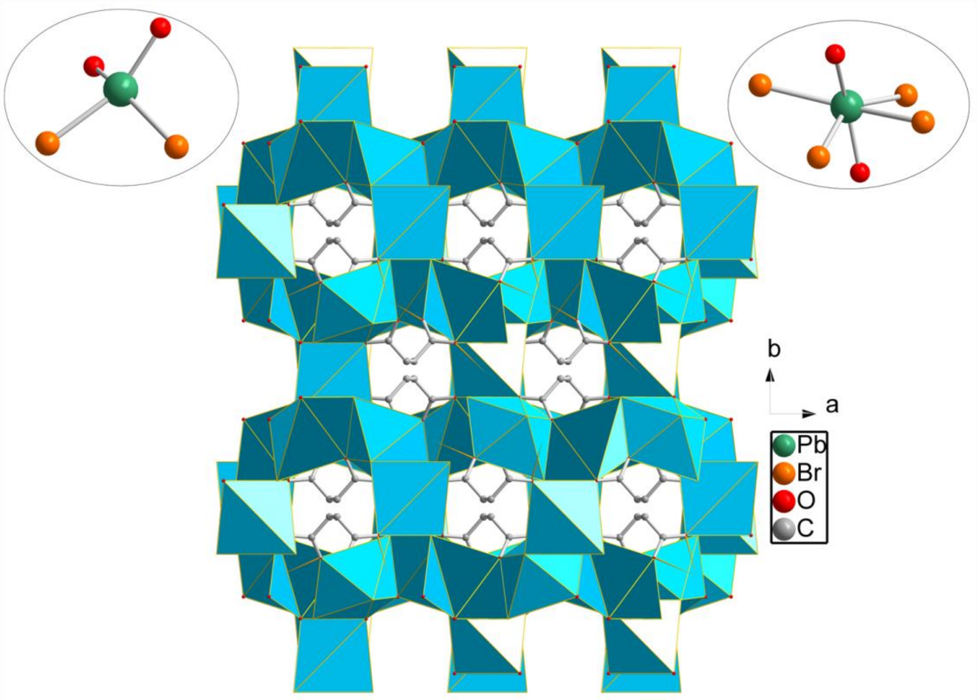

Figure S5. Polyhedral view of the inorganic skeleton of TJU-7 with organic ligands locating in the channels. The insets show the coordination environments of Pb^{II} centers.

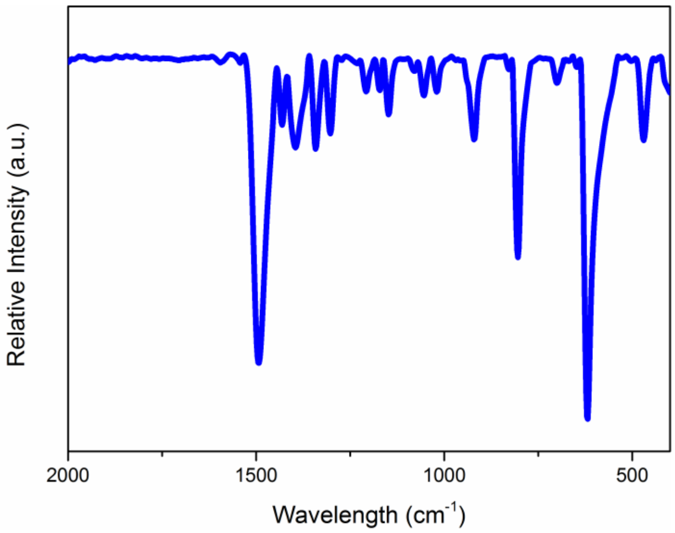

Figure S6. FT-IR spectrum of TJU-6.

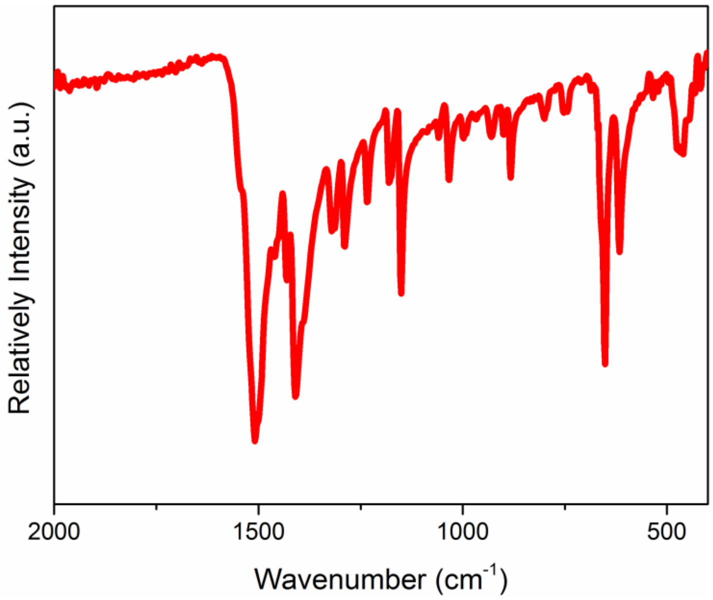

Figure S7. FT-IR spectrum of TJU-7.

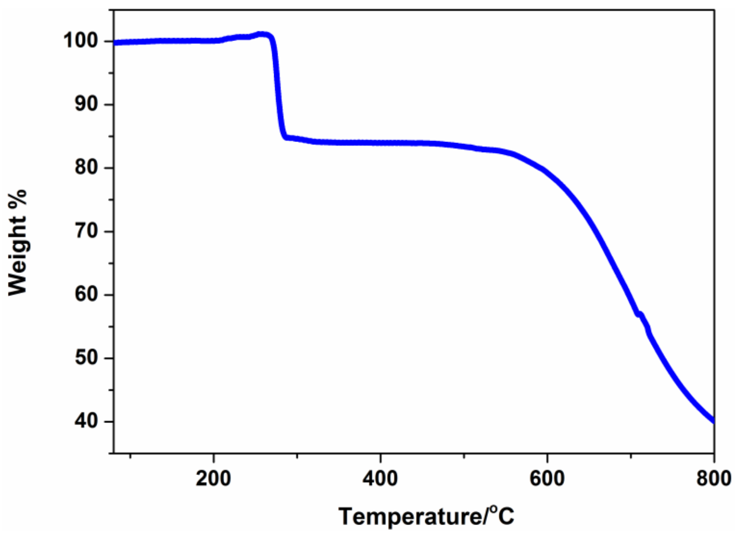

Figure S8. Thermogravimetric analysis curve of TJU-6 in N_2 flow.

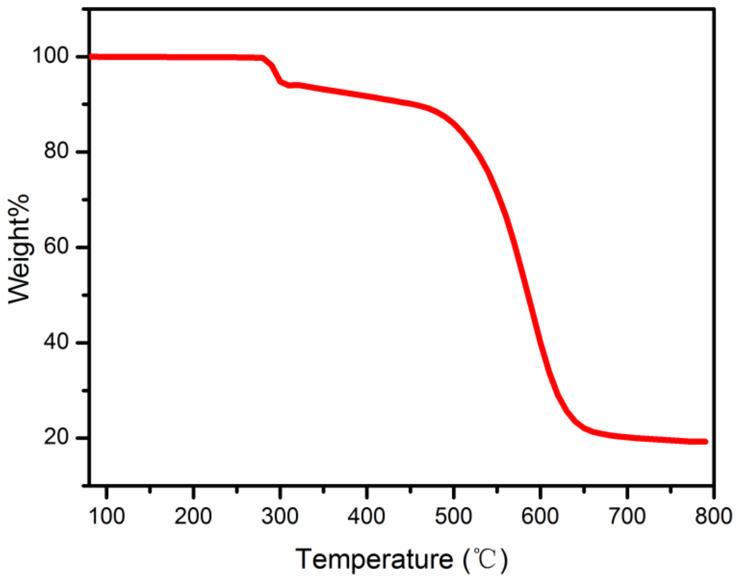

Figure S9. Thermogravimetric analysis curve of TJU-7 in N_2 flow.

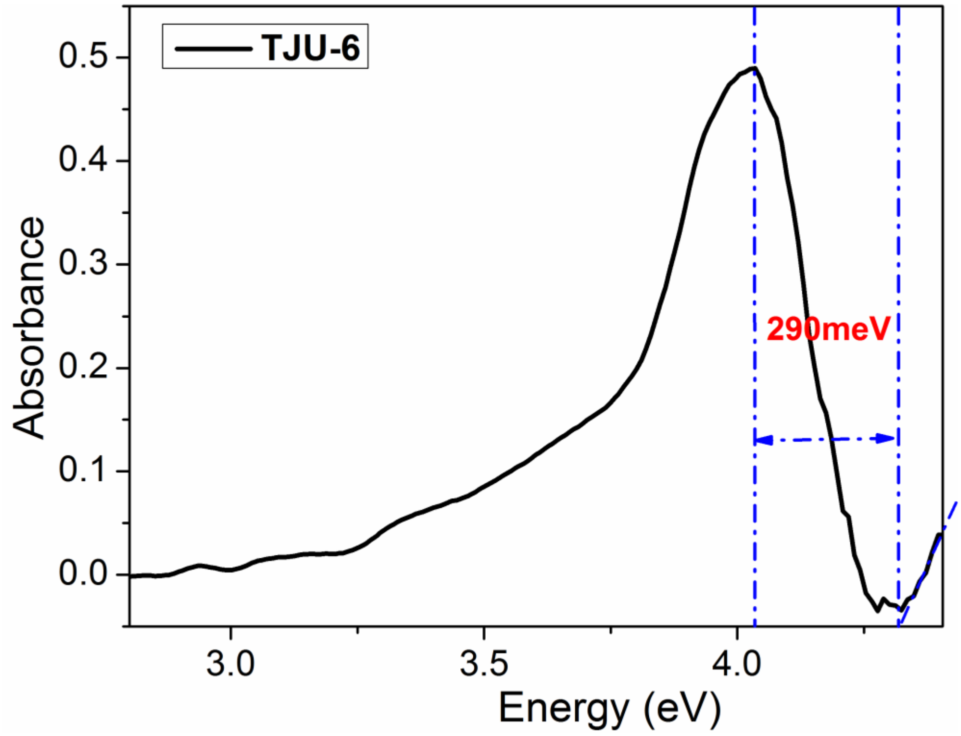

Figure S10. Exciton binding energy determination of TJU-6 at 103 K. The exciton binding energy was estimated taking the difference between the excitonic peak and the onset of the high-energy absorption continuum.

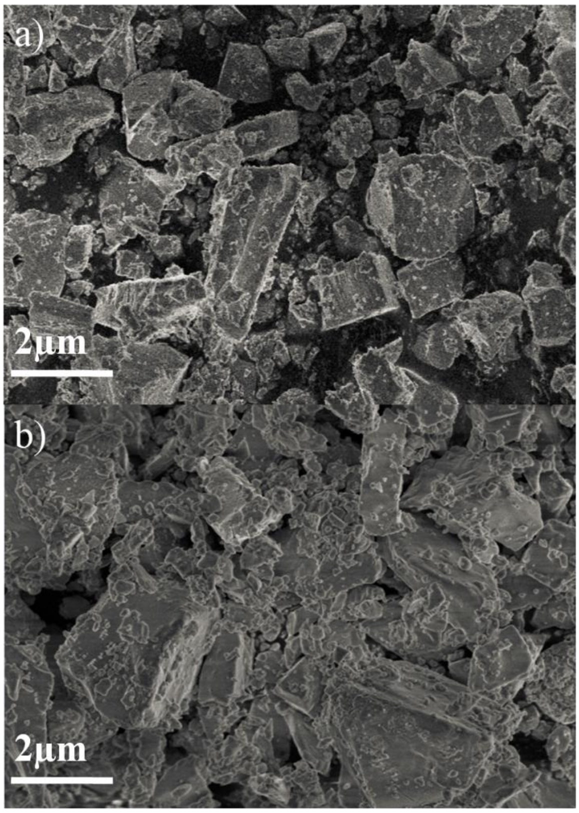

Figure S11. SEM image of μm-sized microscopic powders of TJU-6 (a) and TJU-7 (b).

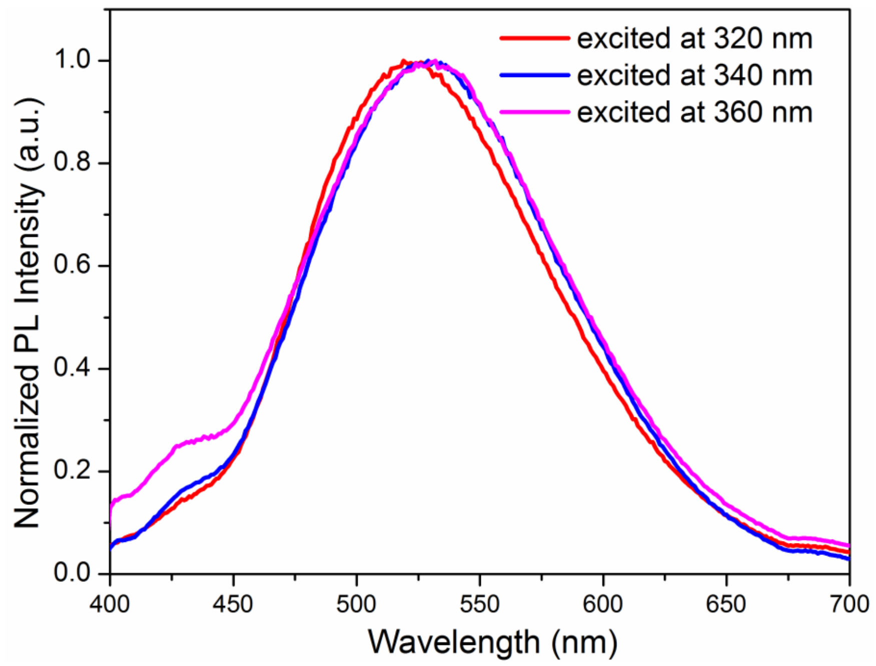

Figure S12. The photoluminescence emission spectras of TJU-6 upon 320 nm, 340 nm and 360 nm excitation.

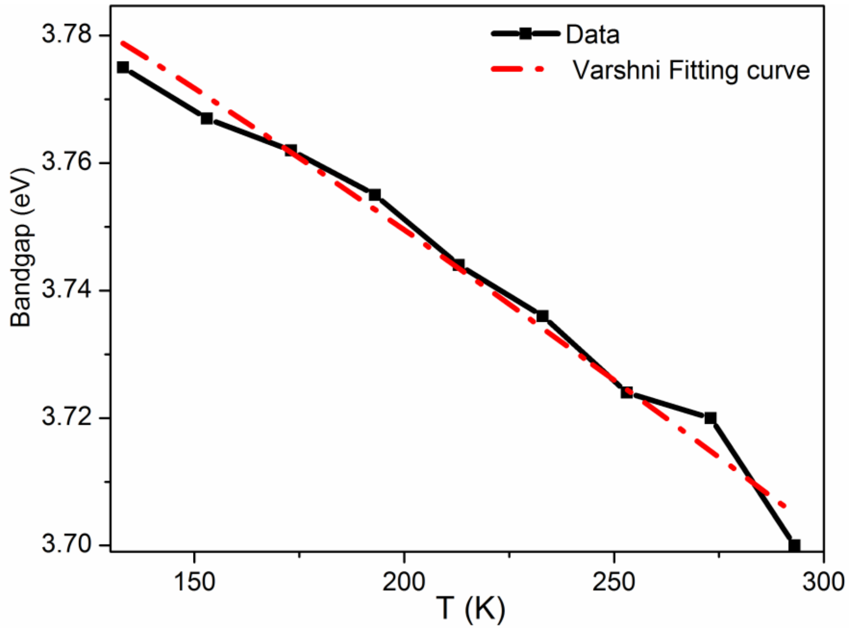

Figure S13. The Varshni's equation fitting of the bandgaps of TJU-6.

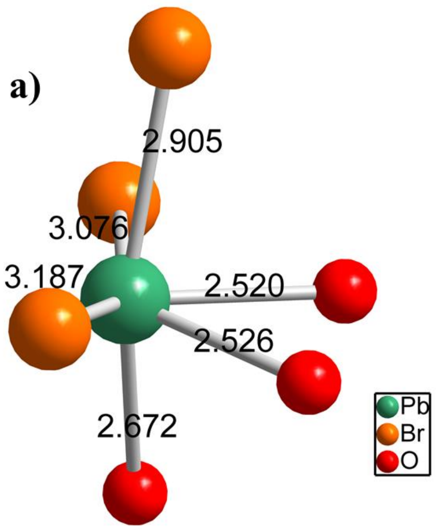

Figure S14. (a) The coordination environment of Pb^{2+} center of TJU-6 (RT). (b) The coordination environment of Pb^{2+} center of TJU-6 (under 150 K).

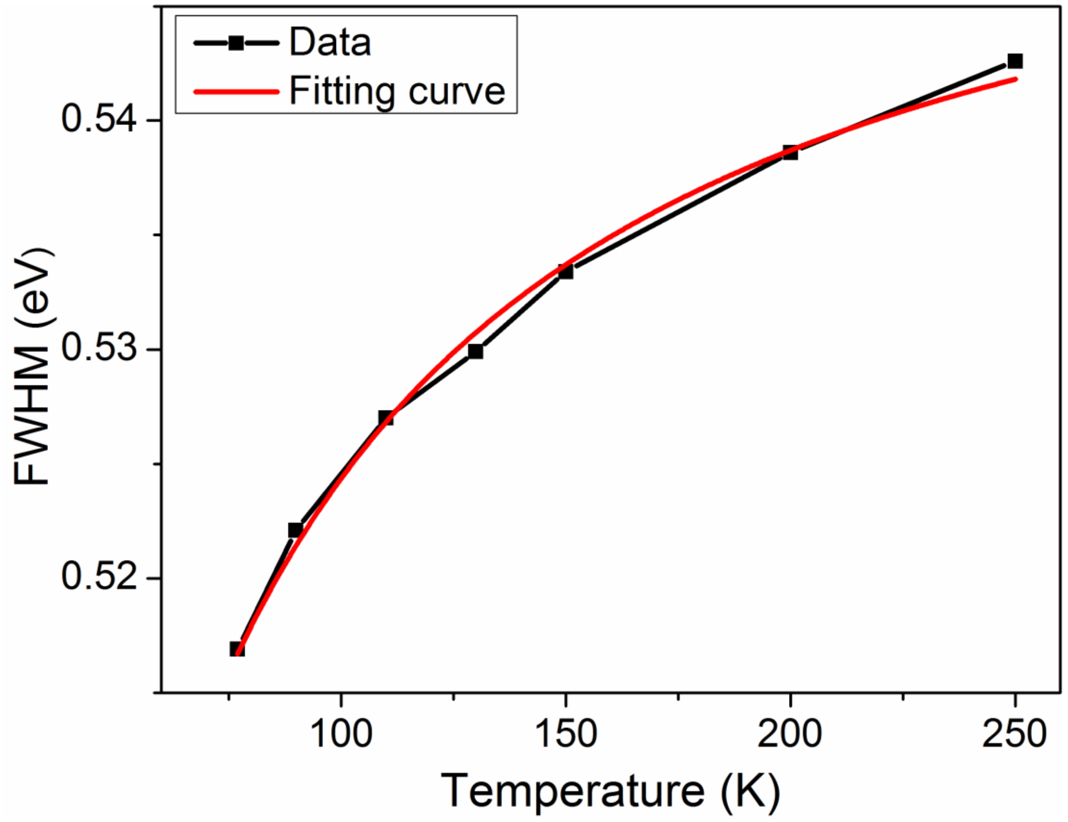

Figure S15. Temperature dependence of the main emission bandwidth in TJU-6 (black symbols) and the best fit (red) to a model (Eq. 3).

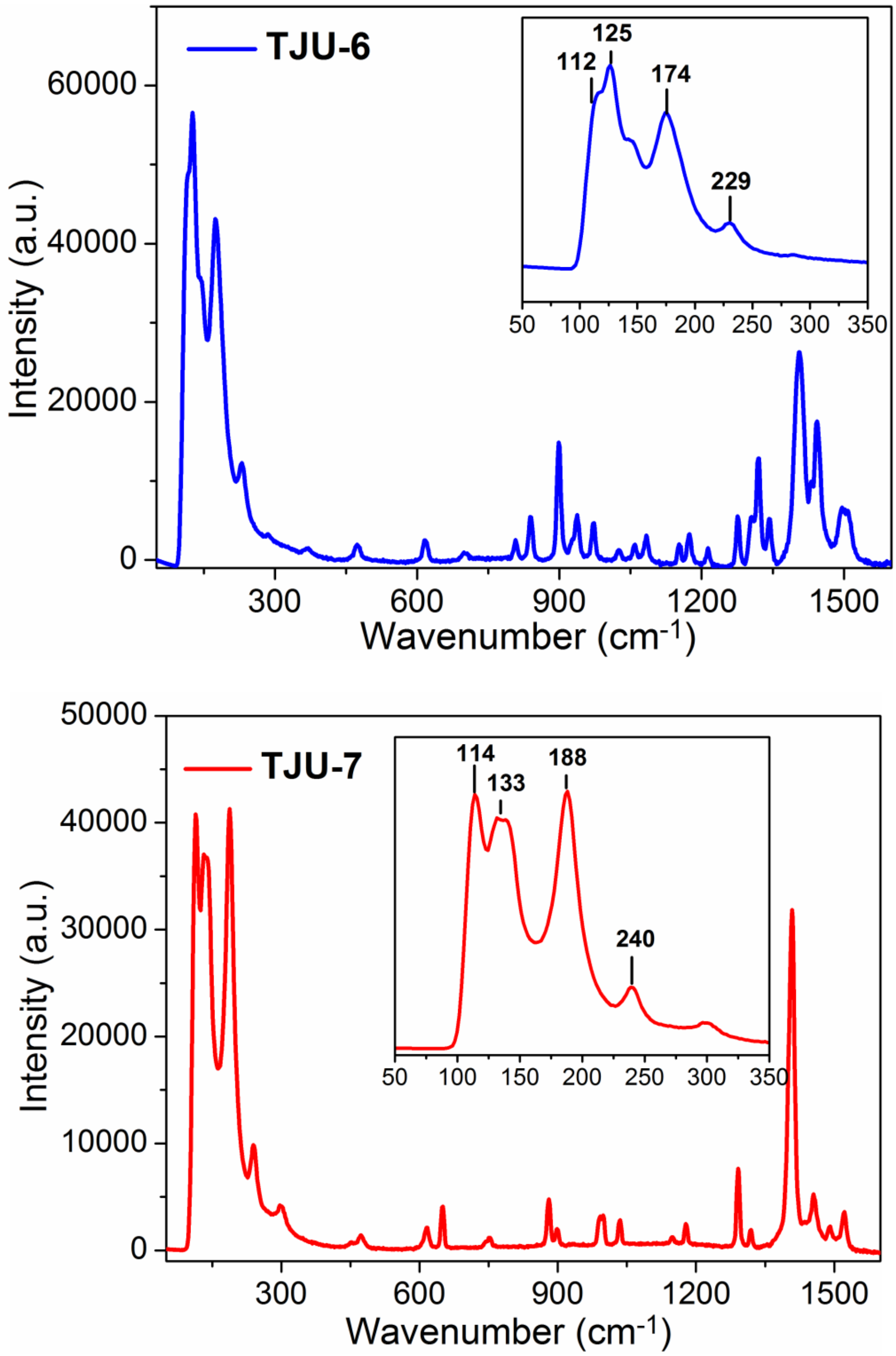

Figure S16. Raman spectra of TJU-6 and TJU-7.

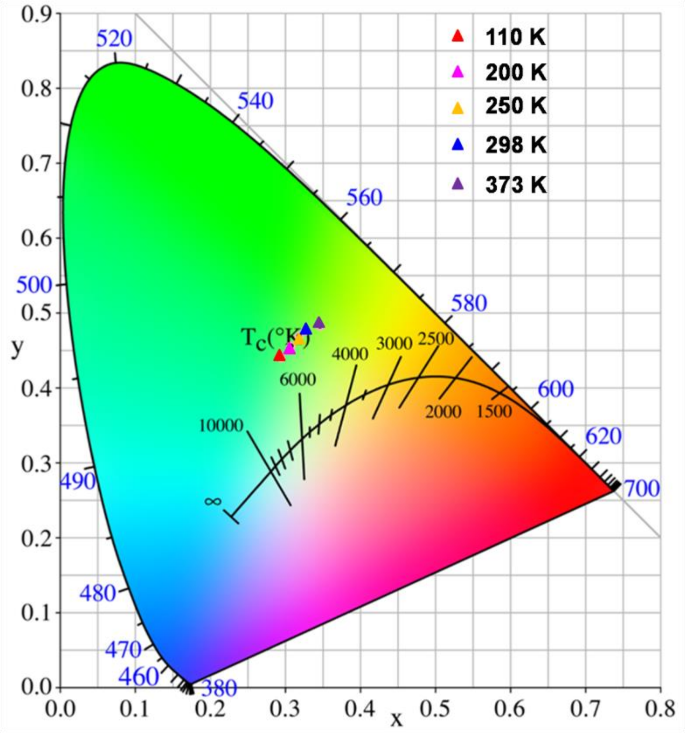

Figure S17. CIE chromaticity coordinates of the TJU-6 from 110 K to 373 K.

References

S1. *APEX-II, 2.1.4*, Bruker-AXS: Madison, WI, 2**007**.

S2. *SHELXTL, Crystal Structure Determination Package*, Bruker Analytical X-ray Systems Inc.: Madison, WI, **1995**, 99.

S3. Brandenburg, K.; Putz, H., Diamond, *Crystal Impact*, Bonn, Germany**, 2007**.

S4. Dohner, E. R.; Hoke, E. T.; Karunadasa, H. I., *J. Am. Chem. Soc.* **2014,** *136* (5), 1718-1721.

S5. Dohner, E. R.; Jaffe, A.; Bradshaw, L. R.; Karunadasa, H. *J. Am. Chem. Soc.* **2014,** *136* (38), 13154-13157.

S6. Thirumal, K.; Chong, W. K.; Xie, W.; Ganguly, R.; Muduli, S. K.; Sherburne, M.; Asta, M.; Mhaisalkar, S.; Sum, T. C.; Soo, H. S., *Chem. Mater.* **2017,** *29* (9), 3947-3953.

S7. Cortecchia, D.; Neutzner, S.; Srimath Kandada, A. R.; Mosconi, E.; Meggiolaro, D.; De Angelis, F.; Soci, C.; Petrozza, A., *J. Am. Chem. Soc.* **2017,** *139* (1), 39-42.

S8. Wang, G.-E.; Xu, G.; Wang, M.-S.; Cai, L.-Z.; Li, W.-H.; Guo, G.-C., *Chem. Sci.* **2015,** *6* (12), 7222-7226.
